# The Utility of a Computerized Algorithm Based on a Multi-Domain Profile of Measures for the Diagnosis of Attention Deficit/Hyperactivity Disorder

**DOI:** 10.3389/fpsyt.2017.00189

**Published:** 2017-10-03

**Authors:** Alessandro Crippa, Christian Salvatore, Erika Molteni, Maddalena Mauri, Antonio Salandi, Sara Trabattoni, Carlo Agostoni, Massimo Molteni, Maria Nobile, Isabella Castiglioni

**Affiliations:** ^1^Child Psychopathology Unit, Scientific Institute, IRCCS Eugenio Medea, Lecco, Italy; ^2^Department of Psychology, University of Milano, Milan, Italy; ^3^Institute of Molecular Imaging and Physiology, National Research Council, Milan, Italy; ^4^Computational Biology Group, Scientific Institute, IRCCS Eugenio Medea, Lecco, Italy; ^5^Pediatric Intermediate Care Unit, Fondazione IRCCS Ca Granda—Ospedale Maggiore Policlinico, DISSCO – Department of Clinical Sciences and Community Health, University of Milan, Milan, Italy

**Keywords:** attention deficit/hyperactivity disorder, machine learning, support vector machines, near-infrared spectroscopy, fatty acids

## Abstract

The current gold standard for diagnosis of attention deficit/hyperactivity disorder (ADHD) includes subjective measures, such as clinical interview, observation, and rating scales. The significant heterogeneity of ADHD symptoms represents a challenge for this assessment and could prevent an accurate diagnosis. The aim of this work was to investigate the ability of a multi-domain profile of measures, including blood fatty acid (FA) profiles, neuropsychological measures, and functional measures from near-infrared spectroscopy (fNIRS), to correctly recognize school-aged children with ADHD. To answer this question, we elaborated a supervised machine-learning method to accurately discriminate 22 children with ADHD from 22 children with typical development by means of the proposed profile of measures. To assess the performance of our classifier, we adopted a nested 10-fold cross validation, where the original dataset was split into 10 subsets of equal size, which were used repeatedly for training and testing. Each subset was used once for performance validation. Our method reached a maximum diagnostic accuracy of 81% through the combining of the predictive models trained on neuropsychological, FA profiles, and deoxygenated-hemoglobin features. With respect to the analysis of a single-domain dataset per time, the most discriminant neuropsychological features were measures of vigilance, focused and sustained attention, and cognitive flexibility; the most discriminating blood FAs were linoleic acid and the total amount of polyunsaturated fatty acids. Finally, with respect to the fNIRS data, we found a significant advantage of the deoxygenated-hemoglobin over the oxygenated-hemoglobin data in terms of predictive accuracy. These preliminary findings show the feasibility and applicability of our machine-learning method in correctly identifying children with ADHD based on multi-domain data. The present machine-learning classification approach might be helpful for supporting the clinical practice of diagnosing ADHD, even fostering a computer-aided diagnosis perspective.

## Introduction

Attention deficit/hyperactivity disorder (ADHD) is among the most common neurodevelopmental disorders, affecting 7.2% of children worldwide (1), with a significant impact on familial, relational, and school functioning in more than one setting. ADHD is a highly heterogeneous condition with manifold causes, progressions and a broad range of symptom manifestations. The diagnostic criteria for ADHD include purely behavioral descriptions of symptoms, which often overlap with the manifestations of many other psychopathologies. Currently, despite the fact that brain structural and functional deficits have been proven in subjects with ADHD, the gold standard for diagnosis consists of subjective measures, such as a clinical interview, observation, and rating scales. These procedures are long term and are heavily based on experiences and practical knowledge of clinicians. The limited reliability of this assessment has led to diagnostic variability across different clinicians and cultures (2). This also contributes to social concerns about the possible harms of misdiagnosing (3, 4).

For these reasons, the diagnosis of ADHD still represents a challenge, and clinicians highly demand the availability of more objective and reliable measures for diagnosing subjects with ADHD. Recent studies have explored the value of behavioral as well as neurophysiological measures for automatically discriminating between children with ADHD and typically developing (TD) peers, possibly fostering a computer-aided diagnosis perspective. These studies typically make use of machine-learning algorithms to distinguish the subjects of different groups by maximizing the distance between datasets. Machine learning commonly refers to all procedures that train a computer algorithm to automatically extract meaningful information from the data and to use it to make predictions about the group membership of new individuals (e.g., patients vs. controls). Machine-learning methods are multivariate analysis methods that also offer the advantage of identifying complex patterns of differences that univariate statistical methods do not efficiently recognize. Thus, the use of these methods should not be simply considered a potential “diagnostic” tool but also a useful procedure for identifying objective measures at an individual level from a larger dataset to be used for single-subject diagnosis. A number of studies recently indicated that the machine-learning classification approach may lead clinicians toward an efficient and accurate diagnosis of ADHD using behavioral/cognitive measures (5) or neurophysiological techniques, such as electroencephalography (6), structural magnetic resonance imaging (MRI) (7), and resting-state functional MRI (8).

In this work, we developed a supervised machine-learning method to recognize school-age children with ADHD and to correctly separate them from TD peers, by using, for the first time, a multi-domain dataset comprising blood fatty acid (FA) profiles, neuropsychological measures, and functional measures obtained from near-infrared spectroscopy (fNIRS). We included these measures based on both the extant literature and on our previous findings. With respect to blood FAs, we followed the suggestion that polyunsaturated fatty acids (PUFAs) shortage could be one of the various etiological factors of ADHD (9), and we recently reported an abnormal FA profile in children with ADHD (10). Furthermore, given the abovementioned work of Bledsoe and colleagues (5) that applied pattern classification methods to behavioral/cognitive measures with promising results, we included the neuropsychological tests of vigilance, attention, and flexibility. Because some debate has occurred in the literature over the use of neuropsychological tests for the diagnosis of ADHD, we were interested in understanding in the present wok the possible predictive value for these measures. Indeed, it was suggested that no psychometric test can be used with confidence for the purposes of diagnostic decision (11, 12) and previous studies demonstrated low to moderate correlations between scores on these tests and other assessment measures for ADHD (13, 14). Finally, such tests do not correlate significantly with parent and teacher ratings of executive functioning (EF) in the child’s daily life activities (15). However, it is worth mentioning that more “ecological” measures of EF could describe different cognitive profiles in children with ADHD-only, ADHD and comorbid disorders, and healthy controls, with a good correlation with parent ratings of EF in everyday activities (16). Finally, we introduced a non-invasive method of functional neuroimaging, fNIRS, to measure the hemodynamic responses to neuronal activation during a spatial working memory task. Near-infrared spectroscopy (NIRS) was chosen among the neuroimaging techniques because it imposes fewer environmental constraints, and due to the low cost of scanning. In addition, previous evidence showed that NIRS could be useful for identifying children with ADHD (17, 18). We included, as stimulation paradigm, a visuospatial *N*-back working memory task consisting of three tasks with increasing difficulty: baseline, 1-back, and 2-back. The choice of such a task was based on the suggestion that a deficit in working memory could be a core cognitive impairment of ADHD (19).

Given that the ADHD etiology is generally considered multifactorial, our hypothesis was that the integration of information from the different sources and levels of analysis would lead to a significantly more effective identification of children with ADHD, compared with using a single-domain dataset per time. Indeed, following a multifactorial etiological model, the emergence of the disorder could be related to the simultaneous malfunction of mechanisms interacting at multiple levels, with each resulting in a different degree of impairment across children. Moreover, our method could support the clinicians’ decision on the tests to be included in the ADHD diagnostic process. In fact, as Kim and colleagues (20) recently proposed in their study predicting methylphenidate response in ADHD *via* the machine-learning approach, we could investigate whether the use of several measures is worth the additional costs of collecting more data, based on any increase in the classification accuracy.

## Materials and Methods

### Participants

Twenty-two children with ADHD were compared with 22 TD children matched by gender, age, and intelligence quotient (IQ). All participants in the clinical group were previously diagnosed according to the Diagnostic and Statistical Manual of Mental Disorders Fourth Edition, text revised (DSM-IV TR) (21) criteria by a medical doctor specialized in child neuropsychiatry with expertise in ADHD (Antonio Salandi and Sara Trabattoni). A child psychologist (Alessandro Crippa) confirmed independently the clinical diagnoses using the semi-structured interview Development and Well-Being Assessment (DAWBA) (22). According to interviews, 18.2% of children in the ADHD group met criteria for the inattentive subtype, 36.4% met criteria for the hyperactive–impulsive subtype, and 45.5% met criteria for the combined subtype. The TD group consisted of children recruited by local pediatricians and from schools in the vicinity of our institute, with no history of medication treatment. Signs of social/communicative disorders and other possible DSM-IV TR diagnoses were excluded in TD children through the administration of the DAWBA interview to parents. To match the two groups, the estimated Full Scale Intelligence Quotient (FSIQ) was measured in TD participants using the Block Design and Vocabulary subtests of the Wechsler Intelligence Scale for Children—III (WISC-III) (23). These two WISC subtests have a correlation of 0.93–095 with the FSIQ (24). Children in both groups were required to have FSIQ or estimated FSIQ scores of higher than 80 on the WISC-III or WISC-IV scales (23, 25). All participants were Caucasian, had normal or corrected-to-normal vision, and were not taking any medication. The study was explained to both children and their parent(s) or caregivers, and all of the participants’ legal guardians signed the informed written consent before the children’s participation. The research received approval from the ethic committee of our institute and was therefore performed in accordance with the ethical standards set forth in the 1964 Declaration of Helsinki and its later amendments.

### Measures

#### Cognitive Profile

The executive function profile of each participant was assessed through a selected battery of cognitive tests from the Amsterdam Neuropsychological Tasks (26). All children completed four computerized tasks, administered in the following order: Baseline speed, Focused attention four letters, Shifting attentional set–visual, and Sustained attention. Baseline speed consisted of a simple reaction time (RT) task. During the Focused attention test, participants had to selectively respond to one target letter among four, when it was presented in the relevant diagonal position, and to ignore it when it was displayed in the irrelevant axis. The Visual set-shifting task was used to investigate three different cognitive dimensions: vigilance, inhibition, and cognitive flexibility. Finally, the Sustained attention was used to assess the fluctuation of attention over time. For further details about the dependent measures considered for these tasks, see Crippa et al. (10).

#### FA Profile

Blood samples were taken from all participants to assess the FA profiles. Samples of blood were directly subjected to transmethylation for gas chromatography analysis, using a well-validated protocol (27). FAs from 14 to 24 carbons were detected. In this study, we reported the percentages of single FAs only for main omega-3 and omega-6. Furthermore, we calculated ratios as arachidonic acid (AA)/eicosapentaenoic acid (EPA) and AA/docosahexaenoic acid (DHA), as they have been pointed out as reliable indexes of the functional effects of long-chain PUFAs (28). Finally, we calculated the sum of EPA and DHA (the “omega-3 index”) (29), and of saturated fatty acid (SFA), monounsaturated fatty acid (MUFA), and PUFA. For further details about the FA profile analysis, see Crippa et al. (10).

Features accounting for both the cognitive and the FA profiles were *Z*-scored according to *z_i_* = (*x_i_* − *m_x_*)/*s_x_*, where *x_i_* is the value of a given feature *x* for the *i*th subject, *m_x_* and *s_x_* are the mean value and the SD, respectively, of feature *x* over the considered population, and *z_i_* is the resulting *Z*-scored feature.

#### Neurophysiological Profile

##### Stimulation Protocol

The stimulation protocol was developed with the Presentation^®^ software (Neurobehavioral Systems Inc.), and stimuli were displayed using a computer screen. The task was a modified version of the visuospatial *N*-back working memory task that Cui and colleagues (30) set up and that lasted approximately 15 min. The paradigm consisted of three tasks—control (C), 1-back (1B), and 2-back (2B)—always presented in this order: rest–C–1B–2B–rest–1B–C–2B–rest–2B–1B–C–rest. The first rest epoch was 60 s long, and the other three rests were 30 s long. During the rest, children passively viewed an image on a black screen. Experimental epochs began with a 3 s display of the instruction: “Repeat” in the 1-back task and “Return” in the 2-back task. Control epochs each began with a 2-s display of the instruction, “Center.” Each control and experimental epoch included 32 stimuli shown for 0.5 s each, with a 1.5 s interstimulus interval. The stimulus was a clown’s face displayed in one of nine locations in a 3 × 3 matrix. In the 1-back task, children were required to respond if the stimulus remained in the same position of the previous trial (“Repeat”). In the 2-back task, participants had to respond whether the stimulus recurred in the same location as it did in the two previous trials (“Return”). In the control task, children were instructed to respond only when the clown’s face was presented in the center of the screen. Practice trials, the number of which varied individually, were given to participants before NIRS recording.

##### NIRS Data Acquisition and Optode Localization

**Figure 1:**
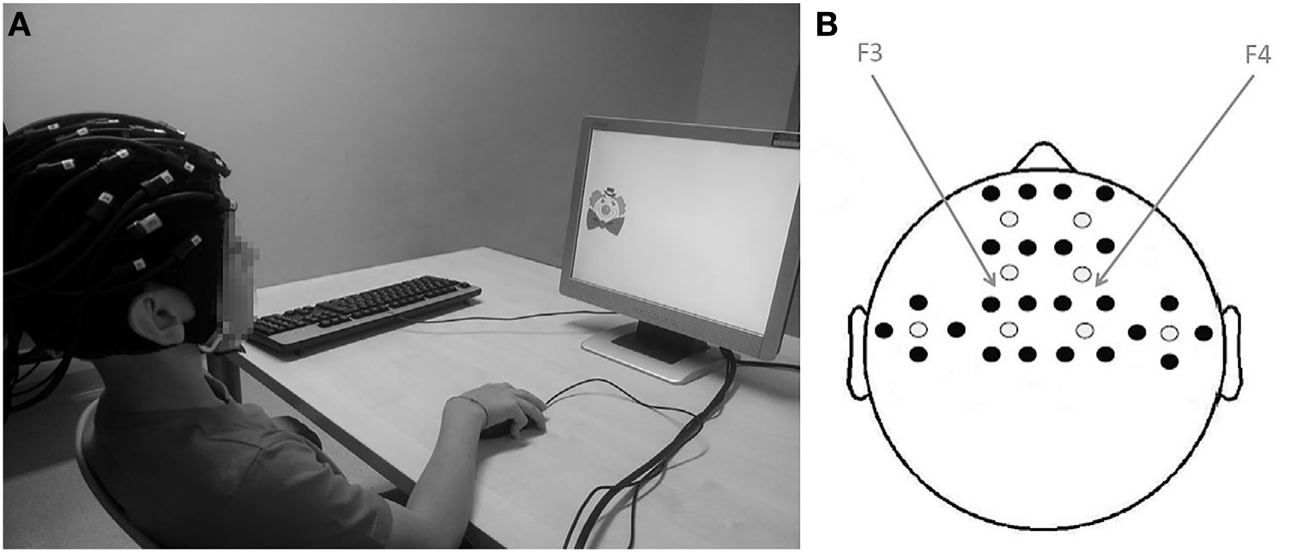
**(A)** Brain activity was measured while participants performed the visuospatial *N*-back working memory task; **(B)** from near-infrared spectroscopy measurements. Location of sources (white dots) and detectors (black dots). The fibers were placed on the bilateral frontotemporal areas centered at F3 and F4 according to the International 10–20 system. The distance between the fibers was set at 2.7 cm.

A commercial continuous wave NIRS device (DYNOT Compact, NIRxBerlin) was employed for NIRS recordings. An elastic cap of the proper head size was fitted on the subject’s head. The cap had 32 channels, with 8 emitters and 24 detectors; it was placed on the child’s scalp at the bilateral frontotemporal areas, centered at F3 and F4 according to the International 10–20 system (31), as shown in Figure 1.

Near-infrared spectroscopy recording was performed at two wave lengths (760 and 830 nm) to probe oxygenated-hemoglobin and deoxygenated-hemoglobin (Deoxy-Hb), respectively, in the brain. The measurement principles were based on the modified Beer–Lambert law, for which oxygenated-hemoglobin and Deoxy-Hb changes are calculated from the change in light attenuation at a given measured point (32).

##### NIRS Preprocessing

First, we visually inspected individual NIRS data to remove obvious artifacts; then, we filtered the signals with a low-pass filter at 0.3 Hz, to respect the task/rest frequency that the stimulation protocol induced. Continuous signals were then divided into epochs starting 14 s before the onset of each task block, and ending 14 s after the end of the blocks. In doing so, nine epochs, lasting 92 s each, were extracted. Epochs were first averaged on the basis of the stimulation type (C, 1B, and 2B) and then grouped, to obtain a grand average. Because the raw NIRS data represented relative values and could not be directly compared across subject or channels, we standardized the raw data into *Z*-scores based on a “baseline” period immediately ahead of the task epoch. In this manner, we could depict the relative change in oxygenated-hemoglobin and Deoxy-Hb concentrations that the visuospatial *N*-back working memory task induced. As Ichikawa and colleagues (11) previously suggested, we selected as “baseline” for standardizing the raw data a period of 3 s just before the beginning of each stimulation period. The *Z*-scores were calculated for each subject *i* using the following formula:

(1) $$z_{i}=(x_{i,\text{task}}-m_{i,\text{baseline}})/s_{i,\text{baseline}},$$

where *x_i_*_,task_ is the raw data (mM mm) of the *i*th subject at each time point during the task period and *m_i_*_,baseline_ and *s_i_*_,baseline_ are the mean value and the SD, respectively, of the raw data of the *i*th subject during the baseline period.

### Data Analysis

First, data were visually and statistically inspected to verify that the assumptions were not violated. A chi-square analysis was then carried out to assess group differences in gender distribution and fish consumption. An independent-samples t test was used to individually examine group differences in age, IQ, and socioeconomic status. The alpha level was set to 0.05 for all data analyses.

### Feature Extraction and Selection

To reduce the features to the most relevant ones for the classification, we performed feature extraction and selection on *Z*-scored data. Feature extraction and selection were applied only to NIRS data.

Feature extraction was performed through principal components analysis (PCA) (33), a technique that has been widely used in the literature across different automatic-classification tasks (34). PCA is able to extract a smaller set of features from the original set of observed data, with these features being referred to as PCA coefficients. PCA coefficients are uncorrelated and sorted according to the maximum-explained-variance criterion.

Extracted PCA coefficients were then ranked according to their Fisher’s discriminant ratios (FDRs); the FDR is an index that measures the binary-class discriminatory power for each feature as follows:

(2) $$\text{FDR}=\frac{{\left(\mu_{\text{ADHD}}-\mu_{\text{TD}}\right)}^{2}}{\sigma_{\text{ADHD}}^{2}+\sigma_{\text{TD}}^{2}},$$

where μ and σ are the mean and the variance, respectively, of the given feature on the whole ADHD or TD dataset. The top 60% PCA coefficients with the highest FDRs were selected and retained for automatic classification.

### The Classifier

A support vector machine (SVM) (35) was used to automatically classify ADHD and TD.

Support vector machines are machine-learning algorithms that able to (1) generate a predictive model by learning how to separate a set of binary-labeled data, called a training set, and (2) use this predictive model to automatically classify unlabeled data in one of the two classes of the training set (in our case, ADHD and TD). The training set consists of (1) a matrix of samples belonging to two different classes, with each sample being represented by a set of selected features, and (2) the corresponding vector of binary labels. Automatic classification was performed using the following features individually: neuropsychological features accounting for the cognitive profile (NPS), features accounting for the FA profile (BIO), features obtained from the oxygenated-hemoglobin NIRS spectra (NIRS OXY), and features obtained from the Deoxy-Hb NIRS spectra (NIRS DEOXY). A classification using NIRS OXY and NIRS DEOXY data taken together was also performed. For each subject, the clinical diagnosis (ADHD and TD) was used as a label for the training of the classifier. The classification system was previously validated in a clinical setting (36). Because we chose to employ a linear kernel (which ensures better computational efficiency in comparison to other kernels), we did not perform any optimization of the SVM regularization hyper parameter.

### Optimization of Features and Performance Evaluation

To find the optimal combination of features for the automatic-classification task, we used a wrapper approach, in which optimization is seen as a search problem. Specifically, different combinations of features were prepared, evaluated through the machine-learning classifier, and assigned a score based on model accuracy. Once all combinations of features were evaluated, the optimal combination was chosen as the one able to return the highest accuracy of classification.

**Figure 2:**
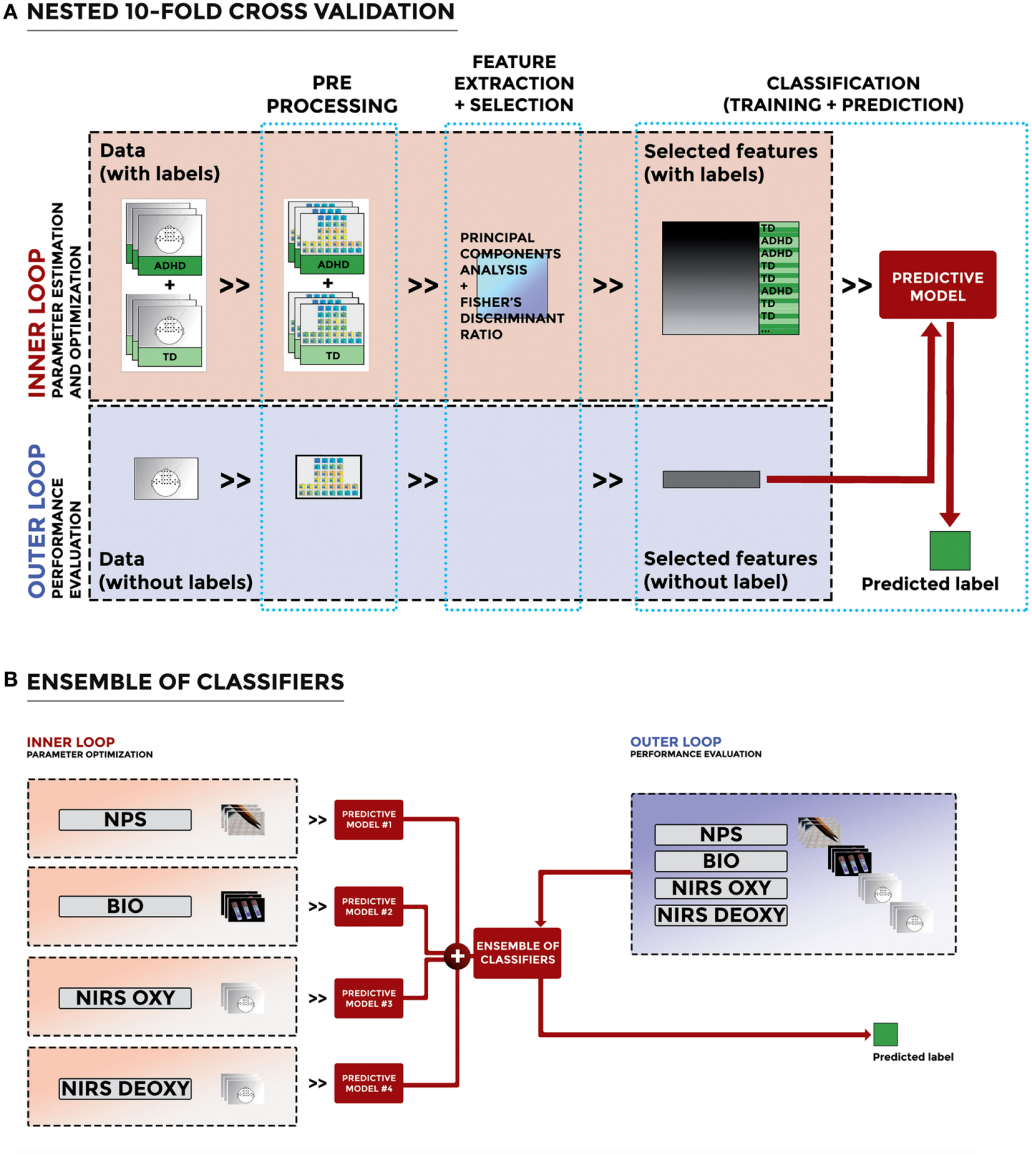
**(A)** Flowchart of the nested cross validation of the proposed machine-learning method for the near-infrared spectroscopy (NIRS) features (as a representative example). The figure shows the different steps of our method, including preprocessing, feature extraction and selection, and classification. **(B)** Flowchart of the ensemble-of-classifiers approach applied to NPS, BIO, and NIRS data.

The optimization of features and performance evaluation were accomplished through a nested 10-fold cross validation (nested CV). The original dataset was split into 10 subsets of (possibly) equal size. Nine out of 10 subsets were used in an inner loop to perform the optimization of features, and the held-out subset was used in an outer loop to evaluate the performance of the classification of the optimal combination of features. This process was repeated 10 times, to use all subsets once for performance evaluation. For each of the 10 rounds, the accuracy, sensitivity, and specificity of classification in the outer loop were evaluated, and the results were averaged across all rounds. The flowchart of the nested CV of the proposed machine-learning method for the NIRS features is depicted in Figure 2A as a representative example.

For cognitive and FA profile data, we were interested in determining which features were the most important for the classification. As mentioned earlier, for each of the 10 rounds of the nested CV, we obtained an optimal combination of features from the inner loop. The importance of features for classification was defined as the occurrence frequency of each feature in the optimal combinations.

### Ensemble of Classifiers

To improve the performance of the single classification systems, we designed an ensemble of classifiers by combining the different predictive models mentioned earlier. Specifically, we trained four different classifiers on independent datasets (NPS features, BIO features, NIRS OXY features, and NIRS DEOXY features, respectively) thus obtaining four independent predictive models, namely the “Predictive model #1” trained on NPS features, the “Predictive model #2” trained on BIO features, the “Predictive model #3” trained on NIRS OXY features, and the “Predictive model #4” trained on NIRS DEOXY features. The predicted outputs were then combined through the majority-vote rule. This procedure allowed us to examine whether the decision of including any additional measure along the diagnostic process is worth the costs, based on any increase in the classification accuracy. Accordingly, given a new (unlabeled) subject, each binary classifier predicts a label for that subject, corresponding to a class. Predicted labels of each binary classifier are then treated as votes for the corresponding class, and the class with the largest number of votes is chosen as the class that the ensemble predicts. A flowchart illustrating this approach is depicted in Figure 2B. The ensemble of classifiers was obtained through the combination of three or four of these predictive models, which led to the following ensembles:

1. NPS + BIO + NIRS OXY
2. NPS + BIO + NIRS DEOXY
3. NPS + NIRS OXY + NIRS DEOXY
4. BIO + NIRS OXY + NIRS DEOXY
5. NPS + BIO + NIRS OXY + NIRS DEOXY

Finally, the proposed classification algorithm was translated into a final software tool that could allow clinicians, by means of a graphic interface, to upload the profile of measures collected from each patient/participant (the blood FA profile, the neuropsychological measures, and the fNIRS spectrum) and to obtain an automatically predicted diagnosis.

## Results

### Participants and Measures

Data on the demographic characteristics of the participants are summarized in Table 1. The statistical analyses confirmed the validity of gender, age, and full-scale IQ matching (all *p* > 0.05). Further, socioeconomic status and weekly fish consumption as referred to by parents were also balanced between groups (both *p* > 0.05).

**Table 1:** Sociodemographic characteristics of the participants.

	ADHD	TD		*p*
*N*	22	22		
Females:males	0:22	1:21	1.023^a^	0.312
Age	11.5 ± 1.5	11.4 ± 1.9	−0.220^b^	0.827
IQ	102.7 ± 11.1	109.6 ± 19.5	1.453^b^	0.154
SES	53.2 ± 20.6	56.1 ± 18.3	0.504^b^	0.617

*ADHD, children with attention deficit/hyperactivity disorder (ADHD); TD, typically developing children; SES, socio economic status; IQ, intelligence quotient*.

*^a^Chi-square test*.

*^b^Student’s t-test*.

### Feature Extraction and Selection

This process was applied only to NIRS data. Feature extraction through PCA resulted in the extraction of *N* − 1 PCA coefficients, where *N* is the number of subjects in the *training* set (*N* = 20 or 21 depending on the particular round of the 10-fold nested CV).

Extracted PCA coefficients were then ranked according to their FDRs. The top 60% (i.e., 12) PCA coefficients with the highest FDRs were selected and retained for automatic classification.

### Optimization of Features and Performance Evaluation

Retained features were fed into the 10-fold nested-CV process for optimization and performance evaluation. Specifically, all NPS (18) and BIO (10) features were used, whereas NIRS features (both NIRS OXY and NIRS DEOXY) were reduced to 12 after feature extraction and selection. Considering that the process was used to evaluate all possible combinations of features, the number of evaluated combinations was as follows:

- NPS: 18 features → 2.6 × 10^5^ combinations
- BIO: 10 features → 1,023 combinations
- NIRS: 12 features → 4,095 combinations

The performances of the machine-learning method for automatically classifying ADHD versus TD are reported in Table 2. Classification was performed using NPS, BIO, NIRS OXY, and NIRS DEOXY features taken individually, as well as NIRS OXY and NIRS DEOXY features taken together. The classification accuracy reached a maximum of 78% (sensitivity 72%, specificity 82%) using NIRS DEOXY features. When using NPS or BIO features taken individually, accuracy resulted in 62 and 66%, respectively.

**Table 2:** Performance of the machine-learning method (accuracy, sensitivity, and specificity) in the automatic classification of attention deficit/hyperactivity disorder vs. typically developing.

Features	Accuracy (mean ± SD)	Sensitivity (mean ± SD)	Specificity (mean ± SD)
NPS	62 ± 17	70 ± 27	57 ± 24
BIO	66 ± 21	58 ± 40	73 ± 29
NIRS OXY	57 ± 27	48 ± 47	67 ± 33
NIRS DEOXY	78 ± 22	72 ± 34	82 ± 24
NIRS OXY + NIRS DEOXY	72 ± 32	73 ± 29	68 ± 43

*Classification was performed for cognitive profile (NPS), fatty acid profile (BIO), features obtained from the oxygenated-hemoglobin NIRS spectra (NIRS OXY), and features obtained from the deoxygenated-hemoglobin NIRS spectra (NIRS DEOXY) taken individually, or NIRS OXY and NIRS DEOXY features taken together. Values of mean and SD were calculated across all the possible folds and round of the cross validation process*.

Besides calculating the performance for the automatic classification of ADHD versus TD, for neuropsychological and biological data, we were interested in determining which features occurred the most in the optimal combinations across the 10 rounds of the nested CV. This provided information about the importance of each feature for the classification. The most discriminant NPS features between the two groups, ranked by occurrence frequency in the optimal combinations, are reported here in descending order: Sustained attention–False alarms, Visual set-shifting–RT inhibition, Sustained attention–Coefficient of variation, Visual set-shifting–Number of inhibition errors, Focused attention–RT correct responses, Focused Attention–Correct rejections target non-relevant positon, Focused attention–SD of correct responses RT, Focused attention–Misses, Sustained attention–Misses, Baseline speed–SD of RT, Visual set-shifting–Number of errors flexibility, Sustained attention–Tempo × Series, Sustained attention–SD, Baseline speed–RT, Focused attention–Correct rejections non-target relevant position, Focused attention–False alarms target non-relevant position, Focused attention–False alarms irrelevant target, Visual set-shifting–RT flexibility. The most discriminant BIO features between the two groups, ranked by occurrence frequency in the optimal combinations, were in descending order: linoleic acid, PUFA, AA, EPA, omega-3 index, AA/DHA, AA/EPA, and MUFA. DHA and SFA were never selected as optimal discriminant features.

### Ensemble of Classifiers

The performances of the ensemble of classifiers for automatically classifying ADHD vs. TD are reported in Table 3. The classification accuracy reached a maximum of 81% [sensitivity 73%, specificity 87%, area under the curve (AUC) 0.80] by combining the predictive models trained on the NPS, BIO, and NIRS DEOXY features. The ensemble of classifiers obtained combining the predictive models trained on the NPS, BIO, NIRS OXY, and NIRS DEOXY features resulted in an accuracy of 76% (sensitivity 83%, specificity 68%, AUC 0.75).

**Table 3:** Performance of the ensemble of classifiers [accuracy, sensitivity, specificity, and area under the curve (AUC)] in the automatic classification of attention deficit/hyperactivity disorder vs. typically developing.

Features				Accuracy (mean ± SD)	Sensitivity (mean ± SD)	Specificity (mean ± SD)	AUC
NPS+	BIO+	NIRS OXY		71 ± 10	70 ± 27	73 ± 24	0.70
NPS+	BIO+		NIRS DEOXY	81 ± 15	73 ± 24	87 ± 22	0.80
NPS+		NIRS OXY+	NIRS DEOXY	78 ± 18	70 ± 36	87 ± 22	0.77
	BIO+	NIRS OXY+	NIRS DEOXY	77 ± 21	63 ± 31	90 ± 21	0.77
NPS+	BIO+	NIRS OXY+	NIRS DEOXY	76 ± 16	83 ± 22	68 ± 23	0.75

**Figure 3:**
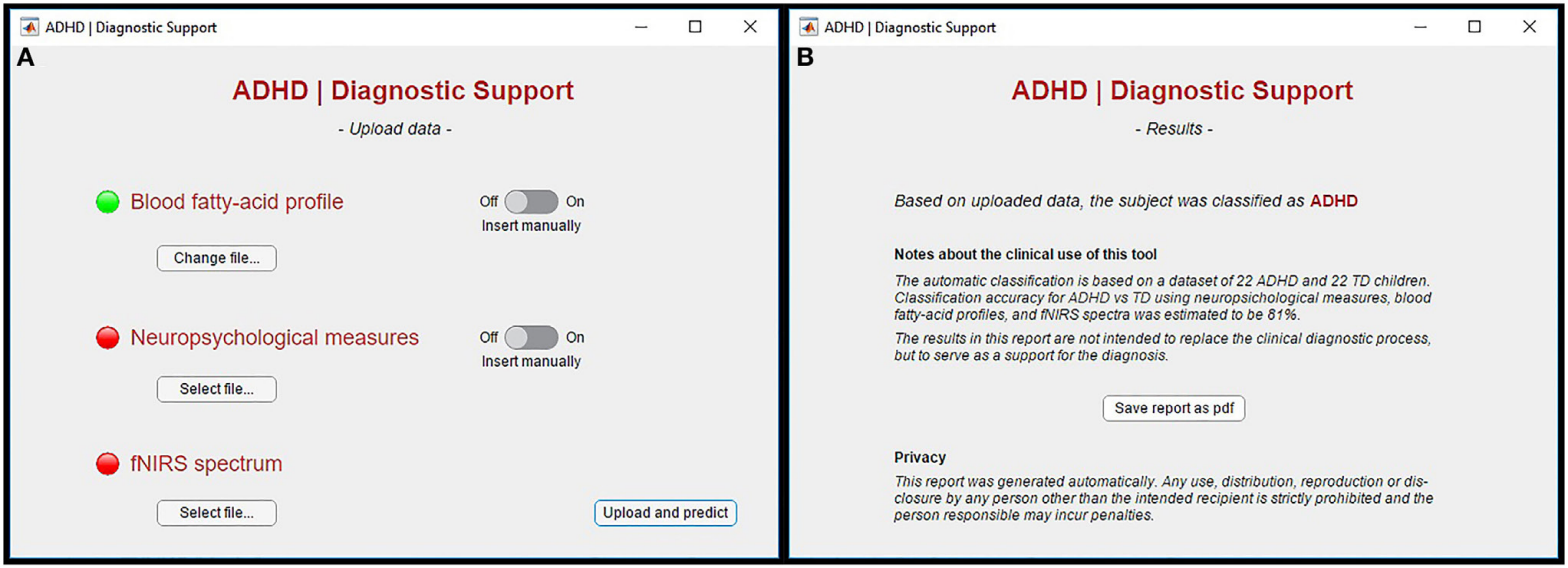
**(A)** Screenshot of the graphic interface developed for the clinical use of the proposed machine-learning method. The tool allows one to upload the expected data *via* a user-friendly interface. Required data are the blood fatty-acid profile, the neuropsychological measures, and the from near-infrared spectroscopy (fNIRS) spectrum of the single patient. **(B)** After uploading the data, the results of the automatic single-subject classification are shown in a new screen, together with some notes about the clinical use of the tool and the privacy.

In Figure 3A, a screenshot of the graphic interface developed for a possible clinical use of the proposed method is shown. Specifically, as it can be seen, the tool allows one to upload the required data *via* a user-friendly interface. Required data are the blood FA profile, the neuropsychological measures, and the fNIRS spectrum of the single patient. After the data are uploaded, the results of the automatic single-subject classification are shown in a new screen, together with some notes about the clinical use of the tool and the privacy (Figure 3B).

## Discussion

In the actual practice, ADHD is diagnosed on the basis of symptoms as judged by clinicians and using qualitative measures, such as a structured interview, rating scales, and neuropsychological tests. The vast heterogeneity of ADHD manifestations represents a challenge for this assessment, potentially leading to misdiagnosis. The goal of this work was to investigate the ability of a multi-domain dataset, including blood FA profiles, neuropsychological measures, and fNIRS measures, to automatically discriminate school-aged children with ADHD from TD peers. To achieve this purpose, we applied for the first time, to our knowledge, a supervised machine-learning method for the analysis of biological, cognitive, and neurophysiological data together to identify children with ADHD. We hypothesized that the integration of information between different levels would increase the classification accuracy of ADHD, as compared with using a single-domain dataset per time. Moreover, we were interested in understanding whether our method could aid in the clinician’s decision about which tests to include in the diagnostic process, based on the trade-off “increase of accuracy/cost.”

With respect to the analysis of a single-domain dataset per time, our machine-learning method reached the lowest individual classification (62%), in the comparisons between children with ADHD and healthy controls, using the neuropsychological data derived from tests of vigilance, focused and sustained attention, and cognitive flexibility. This result was not directly comparable with the previous findings of Bledsoe et al. (5), as the best accuracy reached by the authors and derived from an SVM method also included the behavioral measures of ADHD symptoms, along with the measure of sustained selective attention. Nevertheless, the present result extends the previous findings, suggesting a possible executive/cognitive dysfunction in only 35–50% of children with ADHD (37), and supporting the claim no cognitive test at this time can be used with confidence for diagnostic decision by itself (11, 12). Moreover, the neuropsychological measures that were used to best predict ADHD in our study—false alarms and the intra-subject variability of responses during a sustained attention task, the number of errors, and the response time in a test assessing cognitive inhibition—are among the most frequently reported core cognitive features of ADHD (38, 39). The accuracy of our machine-learning method slightly improved (67%) when we included blood FA values only. Even though the level of classification was still moderate, we believe that this finding could be quite promising if confirmed with larger sample, given the fact that the procedure for collecting these data was fast and minimally invasive and could offer insight on the possible biological signature of ADHD. The present results extend the previous findings of abnormal FA percentages reported in a recent meta-analysis (40), and our previous findings from a clinical study involving a school-aged sample of children with ADHD (10). The FAs that are used to best identify ADHD seem to be linoleic acid, AA, EPA, EPA + DHA, and the total amount of PUFA. All of these components were extensively linked to ADHD in previous studies (40, 41). Our machine-learning method reached greater accuracy (72%) when we used, as a single-domain dataset, the cerebral hemodynamic responses (both oxygenated hemoglobin and deoxygenated hemoglobin) measured by NIRS during a spatial working memory task. Moreover, we achieved an even better accuracy of 78% when we considered only deoxygenated hemoglobin. The classification accuracy that was achieved in this study is nearly consistent with previous SVM applications to fNIRS data (17) or with conservative receiver operating characteristic analysis of fNIRS signal (18). Interestingly, we found a significant advantage of the deoxygenated-hemoglobin over the oxygenated-hemoglobin data (78 vs. 57%) in terms of classification accuracy. The deoxygenated-hemoglobin variation following local neural activation tends to be of a smaller amplitude compared with the oxygenated-hemoglobin response, but it seems to be a more reliable indicator of neural activity, less prone to inference by extra-cerebral physiological noise, and more correlated with the blood oxygenation level dependent signal (42–44). However, this issue is still controversial, with some other works suggesting higher retest reliability for oxygenated hemoglobin (45).

Beyond this, the point of relevance of our work was that, for the first time, we applied a machine-learning approach using biological, cognitive, and neurophysiological data together for automatically classifying children with ADHD. Our ensemble of classifiers reached an accuracy of 76% when we combined the predictive models trained on the whole multi-domain dataset. The classification accuracy of the ensemble reached a maximum of 81% through the combining of the predictive models trained on neuropsychological, FA profiles, and deoxygenated-hemoglobin features. Thus, the present findings clearly show the feasibility and applicability of machine-learning methods in correctly identifying children with ADHD on the basis of multi-domain data. Furthermore, the classification accuracy we reached, using the whole dataset, is consistent with the SVM application of Kim and colleagues (20) to a rich multi-domain dataset (including demographic, clinical, environmental, neuropsychological, neuroimaging and genetic information) to predict the methylphenidate response in ADHD. The performance of the ensemble of classifiers only partially supported our initial hypothesis—i.e., integrating information from different domains would significantly increase the predictive value of our classification approach—as the analysis of the whole dataset minimally improved the classification accuracy (81% for the whole dataset vs. 78% using only NIRS DEOXY signal). Taken together, the results of the ensemble of classifiers clearly showed how the addition of deoxygenated-hemoglobin features increased the accuracy (from 71 to 76–81%). For this reason, we feel that the present findings do not support the decision of including all of the tests we used in a possible diagnostic assessment, on the basis of a gain of accuracy/cost trade-off. In particular, when we considered one domain at a time, we found a limited predictive value for the neuropsychological measures. On the contrary, the significant predictive value of our SVM classification approach of fNIRS data might be valuable for supporting the practice of diagnosing ADHD, even encouraging an fNIRS-based clinical diagnosis at an individual level. Indeed, two other recent studies showed the fNIRS feasibility in identifying children with ADHD with different methodological approaches (17, 18). Moreover, NIRS scanning is relatively low cost. It is also particularly favorable for measuring task-related neural activation in children with ADHD because NIRS requires less stabilization than other neuroimaging techniques do.

Despite our promising results, this study did have some limitations. The first one was that the sample sizes of participant groups were relatively small. To validate the proposed classification approach, we need to replicate the present findings with larger sample sizes so that we can test the computerized algorithm with a totally independent dataset. Another possible limitation of this work was that our classification algorithm was obviously specific to the sample used in training the classifier (i.e., school-aged children with ADHD), so the present findings could not be generalized to adult patients with ADHD. Moreover, future extensions of this study should test the disorder specificity of the classifier, including also other neurodevelopmental conditions frequently associated or in differential diagnosis with ADHD. Keeping these limitations in mind, we clearly emphasize that the proposed technique should not be considered a potential ADHD marker at this time. Indeed, no automatic algorithm can substitute clinical diagnostic decisions based on data from several informants regarding the child’s everyday behavior in different settings. Therefore, in the actual clinical practice, our method should be considered only a complementary and adjunctive one to existent assessment measures. However, the abovementioned limitations should not prevent further investigations using the present approach. In this direction, given the spatial information that NIRS provides, it would be of interest to develop and refine our method to identify which brain regions specifically contribute to the predictive value of the classification.

In conclusion, we have provided preliminary evidence that school-aged children with ADHD can reliably be identified using a multi-domain dataset including blood FA profiles, neuropsychological measures, and fNIRS. The significant predictive value of the present machine-learning classification approach might be helpful for supporting the clinical practice of diagnosing ADHD, even fostering a computer-aided diagnosis perspective.

## Ethics Statement

The study was explained to both children and their parent(s) or caregivers, and all of the participants’ legal guardians gave their informed written consent before the children’s participation. The research was approved by the Ethic Committee of our Institute and has therefore been performed in accordance with the ethical standards laid down in the 1964 Declaration of Helsinki and its later amendments.

## Author Contributions

AC, CS, MMolteni, MN, and IC conceived, designed, and drafted this work; EM conceived and designed the fNIRS paradigm; AS, ST, AC and MMauri performed the clinical and experimental evaluation of all participants; CA critically revised the method for fatty acids’ analysis; all authors critically revised, and approved the final version of and agreed to be accountable for this work.

## Conflict of Interest Statement

The authors have no conflicts of interest to disclose in relation to the specific topic of the present manuscript. The reviewer BD and handling editor declared their shared affiliation.

## Abbreviations

PUFA, polyunsaturated fatty acid; TD, typically developing children; DSM-IV TR, Diagnostic and Statistical Manual of Mental Disorders Fourth edition, text revised; DAWBA, Development and Well-Being Assessment; FSIQ, Full Scale Intelligence Quotient; ANT, Amsterdam Neuropsychological Tasks; FA, fatty acid; AA, arachidonic acid; EPA, eicosapentaenoic acid; DHA, docosahexaenoic acid; SFA, saturated fatty acid; MUFA, monosaturated fatty acid; C, control; 1B, 1-back; 2B, 2-back; F, frontal; Oxy-Hb, oxygenated-hemoglobin; Deoxy-Hb, deoxygenated-hemoglobin; PCA, principal components analysis; FDR, Fisher’s discriminant ratio; NPS, neuropsychological features; BIO, fatty acid profile features; NIRS OXY, Oxy-Hb NIRS features; NIRS DEOXY, Deoxy-Hb NIRS features; CV, cross validation; RT, reaction time; AUC, area under the curve; SVM, support vector machine; BOLD, blood oxygenation level dependent.
